# A Pilot Study on Developing Mucosal Vaccine against Alveolar Echinococcosis (AE) Using Recombinant Tetraspanin 3: Vaccine Efficacy and Immunology

**DOI:** 10.1371/journal.pntd.0001570

**Published:** 2012-03-27

**Authors:** Zhisheng Dang, Kinpei Yagi, Yuzaburo Oku, Hirokazu Kouguchi, Kiichi Kajino, Jun Matsumoto, Ryo Nakao, Hiroyuki Wakaguri, Atsushi Toyoda, Hong Yin, Chihiro Sugimoto

**Affiliations:** 1 Division of Collaboration and Education, Research Center for Zoonosis Control, Hokkaido University, Sapporo, Hokkaido, Japan; 2 State Key Laboratory of Veterinary Etiological Biology, Key Laboratory of Grazing Animal Diseases MOA, Key Laboratory of Veterinary Parasitology of Gansu Province, Lanzhou Veterinary Research Institute, Chinese Academy of Agricultural Sciences, Gansu, People's Republic of China; 3 Department of Biological Science, Hokkaido Institute of Public Health, Sapporo, Hokkaido, Japan; 4 Parasitology Laboratory, School of Veterinary Medicine, Faculty of Agriculture, Tottori University, Tottori, Japan; 5 Laboratory of Medical Zoology, Nihon University College of Bioresource Sciences, Fujisawa, Japan; 6 Department of Medical Genome Science, Graduate School of Frontier Science, The University of Tokyo, Tokyo, Japan; 7 RIKEN Genomic Sciences Center, Yokohama, Kanagawa, Japan; 8 Comparative Genomics Laboratory, National Institute of Genetics, Mishima, Shizuoka, Japan; McGill University, Canada

## Abstract

**Background:**

We have previously evaluated the vaccine efficacies of seven tetraspanins of *Echinococcus multilocularis* (Em-TSP1–7) against alveolar echinococcosis (AE) by subcutaneous (s.c.) administration with Freund's adjuvant. Over 85% of liver cyst lesion number reductions (CLNR) were achieved by recombinant Em-TSP1 (rEm-TSP1) and -TSP3 (rEm-TSP3). However, to develop an efficient and safe human vaccine, the efficacy of TSP mucosal vaccines must be thoroughly evaluated.

**Methodology/Principal Findings:**

rEm-TSP1 and -TSP3 along with nontoxic CpG ODN (CpG oligodeoxynucleotides) adjuvant were intranasally (i.n.) immunized to BALB/c mice and their vaccine efficacies were evaluated by counting liver CLNR (experiment I). 37.1% (*p*<0.05) and 62.1% (*p*<0.001) of CLNR were achieved by these two proteins, respectively. To study the protection-associated immune responses induced by rEm-TSP3 via different immunization routes (i.n. administration with CpG or s.c. immunization with Freund's adjuvant), the systemic and mucosal antibody responses were detected by ELISA (experiment II). S.c. and i.n. administration of rEm-TSP3 achieved 81.9% (*p*<0.001) and 62.8% (*p*<0.01) CLNR in the liver, respectively. Both the immunization routes evoked strong serum IgG, IgG1 and IgG2α responses; i.n. immunization induced significantly higher IgA responses in nasal cavity and intestine compared with s.c. immunization (*p*<0.001). Both immunization routes induced extremely strong liver IgA antibody responses (*p*<0.001). The Th1 and Th2 cell responses were assessed by examining the IgG1/IgG2α ratio at two and three weeks post-immunization. S.c. immunization resulted in a reduction in the IgG1/IgG2α ratio (Th1 tendency), whereas i.n. immunization caused a shift from Th1 to Th2. Moreover, immunohistochemistry showed that Em-TSP1 and -TSP3 were extensively located on the surface of *E. multilocularis* cysts, protoscoleces and adult worms with additional expression of Em-TSP3 in the inner part of protoscoleces and oncospheres.

**Conclusions:**

Our study indicated that i.n. administration of rEm-TSP3 with CpG is able to induce both systemic and local immune responses and thus provides significant protection against AE.

## Author Summary

Humans and rodents become infected with *E. multilocularis* by oral ingesting of the eggs, which then develop into cysts in the liver and progress an endless proliferation. Untreated AE has a fatality rate of >90% in humans. Tetraspanins have been identified in *Schistosoma* and showed potential as the prospective vaccine candidates. In our recent study, we first identified seven tetraspanins in *E. multilocularis* and evaluated their protective efficacies as vaccines against AE when subcutaneously administered to BALB/c mice. Mucosal immunization of protective proteins is able to induce strong local and systemic immune responses, which might play a crucial role in protecting humans against *E. multilocularis* infection via the intestine, blood and liver. We focused on Em-TSP3, which achieved significant vaccine efficacy via both s.c. and i.n. routes. The adjuvanticity of nontoxic CpG OND as i.n. vaccine adjuvant was evaluated. The widespread expression of Em-TSP3 in all the developmental stages of *E. multilocularis*, and the strong local and systemic immune responses evoked by i.n. administration of rEm-TSP3 with CpG OND adjuvant suggest that this study might open the way for developing efficient, nontoxic human mucosal vaccines against AE.

## Introduction

Alveolar echinococcosis (AE), caused by *E. multilocularis*, is known as a very important zoonotic disease, which is endemic in the large areas of the Northern Hemisphere [1] and is often life-threatening. *E. multilocularis* infection of intermediate hosts (humans and rodents) occurs after oral ingestion of mature oncosphere-containing eggs. In the small intestine, the oncospheres hatch out and then migrate via the hepatic vein to the liver, where they form cyst masses and increasingly transform into multiple vesicles filled with fluid and protoscoleces. The parasitic vesicles are lined with a germinal layer (GL) and a laminated layer (LL), which are immediately surrounded by an exuberant granulomatous response generated by the host immune system [2], [3]. Development/infection of *E. multilocularis* larvae in host intestine, blood and liver is characterized by systemic and/or mucosal immune responses. However, it doesn't mean that all the immune responses are protection-associated. To the contrary, some are modulated by the parasites and are thus susceptibility-associated. In particular, during the chronic stage of infection, protective immune responses are down-regulated by *Echinococcus* parasites using some molecules for benefiting their long-term survival in the intermediate host liver [4]–[7]. Studies of immunological profiles showed that, in the infected intermediate host, early Th1-polarized cytokine production, which can kill the metacestodes at the initial stages of development, shifts to a predominantly Th2 response during the chronic stage [4], [6], [8]. It is believed that in *Echinococcus* infection, Th2 responses are mainly associated with susceptibility to *Echinococcus* infection, whereas Th1 responses contribute to protection [5], [6], [8]–[13]. As was shown, some of the proteins expressed on the surface of, or excreted by cestode parasites are involved in immunoregulations, whereby the parasites escape host immune attack and survival in the long term [14]–. Therefore, suppressing/interfering with the function of these proteins using specific antibodies or immune-associated cytokines are key points considerable for efficient vaccine design. Much progress has been made in vaccine development against *Schistosoma* parasite infection using a surface protein, tetraspanin [15]–[17]. In our previous study, seven tetraspanins have been identified in *E. multilocularis* larvae and are used to develop vaccines against *E. multilocularis* infection, which induced significant levels of protection when subcutaneously administered with Freund's adjuvant [18]. Remarkably, vaccinations with rEm-TSP1 and -TSP3 were shown to induce strong serum IgG immune responses in immunized BALB/c mice and received an >85% of liver cyst lesion number reductions (CLNR) after orally challenged with parasite eggs. However, due to the toxicity of Freund's adjuvant [19], [20], an extensive application of this vaccine model in humans is not feasible.

Of the adjuvants used to develop anti-helminth vaccines, CpG ODN has been proved an ideal choice for its non-toxicity and ability enable to induce strong systemic and/or local protective immune responses [21]. Many studies developing anti-protozoan and -helminth vaccines used CpG as an adjuvant [22]–[24]. Evaluation of the adjuvant efficacy and safety of CpG in primates, including humans [25], [26], made it possible for developing safe human vaccines.

CpG was reported to induce strong anti-parasite mucosal immune responses [22]. Mucosal administration is painless and easier than other administration routes and able to induce more specific antibodies, predominantly in local secretions, against pathogens invasion [27]. Intranasal administration (i.n.), the most efficient mucosal delivery route for antigens, has the following properties that make it the priority route in the present study. First, it is thought to confer the highest level of mucosal immunity, which is capable of priming a full range of local immune responses (so-called ‘common mucosal immune system’) as well as systemic immune responses against protective antigens [28], and only requires a small antigen dose [29], [30]. Second, it does not require injection and is therefore safe and painless. Third, it does not require trained medical personnel for delivery and is thus more appropriate for mass vaccination programmes, especially in under-developed countries [27]. To date, i.n. immunization of antigens against helminth infection has achieved much in *Ascaris suum* [31], [32], *Trichinella spiralis* [33], *Schistosoma mansoni* [34] and *E. granulosus* [35].

As a pilot study on evaluation of i.n. vaccine efficacy of TSPs, two independent experiments were performed by us. In experiment I, we compared the i.n. vaccine efficacy between rEm-TSP1 and -TSP3 which showed >85% of liver CLNR in our previous study, when used as an s.c. vaccine [18]. In experiment II, we evaluated the vaccine efficacy of rEm-TSP3 plus Freund's adjuvant (s.c.) versus rEm-TSP3 plus CpG adjuvant (i.n.) under the same experimental conditions. The systemic (serum) and local (nasal cavity, intestine and liver) immune responses induced by both administration routes were investigated and their possible roles in protection discussed.

## Materials and Methods

### Ethics statement

This study was carried out in strict accordance with the recommendations set out in the Guidelines for Animal Experimentation of the Japanese Association for Laboratory Animal Science and the protocol for the animal experiments was approved by the ethics committee of Hokkaido University (Permit Number: 09-0144) and the Hokkaido Institute of Public Health (Permit Number: K20-6). All surgery was performed under isoflurane anesthesia, and all efforts were made to minimize suffering.

### Cloning and expression of Em-TSP1 and Em-TSP3

The regions encoding the LEL (large extracellular loop) domain of Em-TSP1 and Em-TSP3 were amplified from the full-length enriched cDNA library of *E. multilocularis* metacestode, subcloned into the pBAD/Thio-TOPO vector (Invitrogen, USA), expressed and purified as previously described [18]. Briefly, *E. coli* TOP10 cells (Invitrogen, USA) were transformed with a recombinant plasmid according to the manufacturer's instructions (pBAD/TOPO® ThioFusion™ Expression Kit, Invitrogen, USA). Recombinant proteins were purified from *E. coli* lysates using a HisTrap affinity column under nondenaturing conditions (HisTrap FF crude 1 ml, GE Healthcare, USA) and stored at −80°C. A pBAD/Thio-TOPO vector without inserts was also expressed and thioredoxin (TRX) was purified as a negative control. Genbank accession number for tetraspanins used in this study and those referred in the text were listed in Table S1.

### Localization of Em-TSP1 and Em-TSP3 by immunohistochemistry

Cyst tissue and protoscoleces of Hokkaido isolate were derived individually from infected Mongolia gerbils and cotton rats. Adult worms were isolated from the intestine of an infected dog. Samples were transferred to freezing medium (Tissur-O.C.T Compound, Miles, USA) and stored at −80°C. Cryosections (5 µm) were cut on a Leica CM1900 rotatory microtome (Leica, Germany) at −20°C, mounted on slides, and then fixed in acetone for 10 min. After air drying, the slides were re-hydrated in phosphate buffered saline (PBS) and endogenous peroxidase was inactivated by incubating for 10 min in 0.3% hydrogen peroxide (H_2_O_2_) (in methanol). Samples were washed with PBS for 5 min and incubated with rabbit anti-rEm-TSP1 and -TSP3 antibodies respectively, at a dilution of 1∶600 in 3% BSA/PBS for 1 h. After an additional washing step as above, the slices were incubated with Alexa Fluor R488 goat anti-rabbit IgG (H+L) (Invitrogen, USA) at a dilution of 1∶2,000 in 3% BSA/PBS for 1 h. Following three washing steps, the stained samples were embedded in glycerol/phosphate buffer (v/v, 9∶1) and viewed under an Olympus BX50 fluorescence microscope (Olympus, Japan). All procedures were carried out at room temperature. Pre-immune rabbit serum was used as a negative control.

### Mice and parasite eggs

Five-week-old BALB/c mice (male) were maintained in cages in a P3 animal room at 23–25°C with a 12 h light/12 h dark cycle. Litter was cleaned weekly. They were provided with food and water ad libitum and immunized at the age of 6 weeks. *E. multilocularis* (Hokkaido isolate) eggs were collected from the feces of an experimentally-infected dog. Eggs were microscopically observed to confirm their morphological integrity before challenge (classical morphology of an egg isolated from the dog is shown in Figure S1).

### Experimental design

#### Experiment I: Efficacy of i.n. rEm-TSP1 and -TSP3 vaccines

To determine the vaccine efficacy of rEm-TSP1 and -TSP3 administered i.n., 25 BALB/c mice were divided into five groups (Table 1). Mice were immunized once per week for 3 weeks with rEm-TSPs at a dose of 50 µg/animal (diluted in 50 µl PBS) with adjuvant CpG ODN (1 nM/mouse) (Hokkaido System Science, Japan). CpG ODN containing unmethylated CpG motifs acts as immune adjuvant in mice, boosting the humoral and cellular response to coadministered antigens. One week after the final immunization, mice were anesthetized and challenged orally with 0.5 ml *E. multilocularis* eggs (400 eggs/ml in physiological saline) collected from the feces of an experimentally-infected dog. One month post-infection, all mice were sacrificed and necropsies performed for counting the number of cyst lesions (Figure S2).

**Table 1:** Intranasal vaccination groups used for experimental I.

Group	Protein (50 µg/mouse)	Number of mice	Eggs for challenge
1	PBS	5	200
2	PBS+CpG	5	200
3	TRX+CpG	5	200
4	rEm-TSP1+CpG	5	200
5	rEm-TSP3+CpG	5	200

#### Experiment II: Efficacy of the s.c.- and i.n.-administered rEm-TSP3 vaccine and associated immune responses. 1. S.c. and i.n. administration

To confirm the vaccine efficacies of rEm-TSP3 delivered via the i.n. and s.c. administration routes and to examine the protection-associated immune responses, 72 BALB/c mice were grouped as shown in Table 2. The recombinant protein, rEm-TSP3 (which produced the highest reduction in the number of cyst lesions in experiment I) was weekly immunized to BALB/c mice at a dose of 50 µg/mouse (in 50 µl PBS) plus CpG adjuvant (1 nM/mouse) (i.n.) or Freund's complete adjuvant (CFA, MP Biomedicals, USA) and Freund's incomplete adjuvant (IFA, MP Biomedicals, USA) (s.c.) for three times. Freund's adjuvant was used at a dose of 50 µl per mouse individually to minimize the suffering to mice (http://oacu.od.nih.gov/ARAC/documents/Adjuvants.pdf). After the last immunization, blood was collected from the mice orbits using glass capillary pipettes (Hirschmann, Germany) and the serum was separated. Three out of eight mice from each group were sacrificed, and the nasal cavity washes, intestines and liver were collected. Samples were stored at −20°C. Challenge with parasite eggs and the procedures used to count the number of cyst lesions in the remaining mice were conducted as described in experiment I.

**Table 2:** Subcutaneous and intranasal vaccination groups in experiment II.

Group (s.c.)	Protein (50 µg/mouse)	Number of mice	Eggs for challenge
1	PBS	8	200
2	PBS+CFA/IFA	8	200
3	TRX+CFA/IFA	8	200
4	rEm-TSP3+CFA/IFA	8	200

Group (i.n.)	Protein (50 µg/mouse)	Number of mice	Eggs for challenge
5	PBS	8	200
6	PBS+CpG	8	200
7	TRX+CpG	8	200
8	rEm-TSP3	8	200
9	rEm-TSP3+CpG	8	200

s.c. = subcutaneous;

i.n. = intranasal.

#### 2. ELISA (enzyme linked-immunosorbent assay) determination of antibody responses

To evaluate the IgG (including IgG1 and IgG2α subclasses), IgA and IgM antibody responses in blood induced by vaccination via the two administration routes used in experiment II, bloods were collected and sera separated. To evaluate IgA antibody responses in the local mucosal, nasal cavity washes were collected in 500 µl of PBS (pH 7.4) and insoluble debris removed by centrifugation. A 10-cm fragment of the ileal region of the intestine was excised and the intestinal tube was opened using scissors and immersed in 250 µl of PBS. Livers were homogenized in 500 µl of PBS and extracts were obtained by centrifugation. Samples were vigorously vortexed and centrifuged to collect the supernatants. Indirect ELISA was performed for the antibody analysis as previously described [18]. Briefly, 96-well microtiter plates (Corning, USA) were coated with rEm-TSP1/rEm-TSP3 proteins (0.25 µg/well), blocked with 5% skim milk. To detect serum IgG, IgG1, IgG2α, IgA and IgM, plates were incubated with sera at a dilution of 1∶2,000 followed by incubation with horseradish peroxidase (HRP)-conjugated anti-mouse IgG (Invitrogen, USA), IgG1 (Rockland, USA), IgG2α (SouthernBiotech, USA), IgA (Invitrogen, USA) or IgM (MP Biomedicals, USA). To detect IgA responses in nasal cavity, intestine and liver, plates were incubated with the nasal cavity washes, intestine washes and liver extracts at a dilution of 1∶10, respectively, followed by incubation with HRP-conjugated anti-mouse IgA (Invitrogen, USA). Color reactions were developed by addition of 100 µl of TMB (3, 3′, 5, 5′-tetramethylbenzidine) substrate (Dojindo, Japan). Absorbance was measured at 450 nm in a Biotrak II plate reader (Amersham Biosciences, USA). Antisera were pre-absorbed with purified TRX to deplete antibodies directed to the fusion partner protein TRX.

#### 3. Determination of complement-mediated lysis of protoscoleces *in vitro*

Sera from rEm-TSP3-immunized and -non-immunized mice were collected after the third immunization. Protoscoleces were obtained from a cotton rat orally infected with eggs 6 months at the Hokkaido Institute of Public Health. The number of protoscoleces was adjusted to 1,000/ml in PBS and 0.025 ml (about 25 protoscoleces) was added to a concave slide. Untreated/normal serum and serum incubated at 56°C for 30 min (complement inactivation) were used to confirm the lytic effect of complement. To identify the complement activation pathway involved, sera were incubated at 50°C for 30 min to inactivate C3 proactivator. Sera (0.05 ml) were added to the prepared protoscoleces on the concave slide and mixed before a cover slip was applied. Concave slides were incubated at 37°C and protoscolex lysis was observed under light microscope after 0.5, 1, 2 and 8 h.

#### 4. Evaluation of the protective efficacy of the rEm-TSP3 vaccine

One month post-infection, all mice were sacrificed and necropsies performed. Livers were collected in plastic dishes and stored at 4°C overnight to harden them before they were cut into approximately 0.5-mm thick slices (Figure S2). All detectable lesions were counted and the protection rate (percentage reduction in the cyst lesions of experimental groups compared with that in the PBS control) was calculated using the formula:
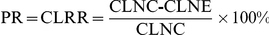
where PR = protection rate, CLRR = cyst lesion reduction rate, CLNC = cyst lesion number from control group, CLNE = cyst lesion number from experimental group.

### Statistical analyses

The data were analyzed using one-way ANOVA followed by a multiple comparison Tukey's test. Differences were considered statistically significant at *p*<0.05, very significant at *p*<0.01 and extremely significant at *p*<0.001.

## Results

### Immunolocalization of Em-TSP1 and Em-TSP3 in larval and adult *E. multilocularis*

**Figure 1:**
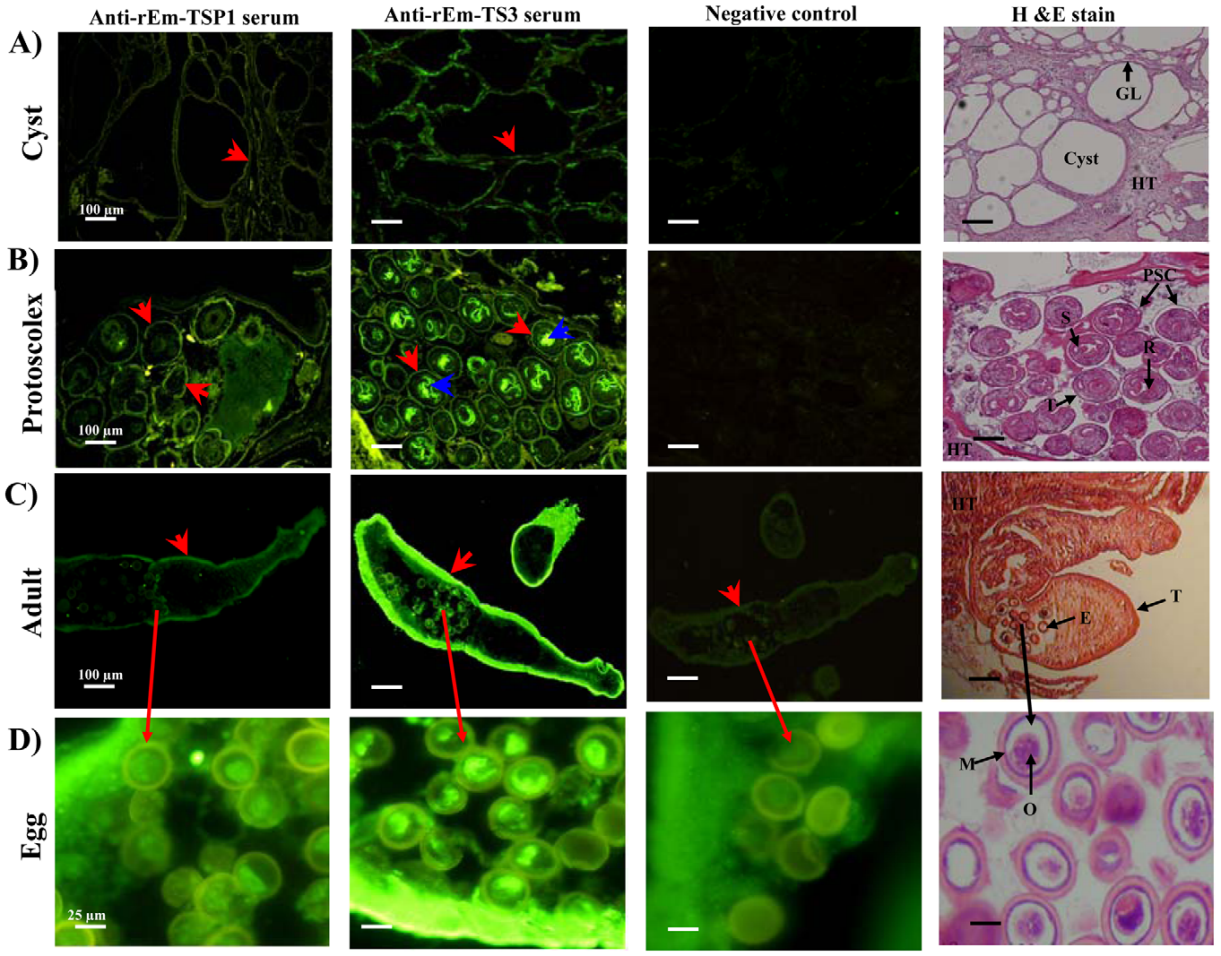
Immunolocalization of Em-TSP1 and Em-TSP3 on the larval and adult stages of *E. multilocularis*. Polyclonal rabbit thioredoxin-absorbed anti-Em-TSP1 and anti-Em-TSP3 antibodies, pre-immunized sera (negative controls), and Alexa Fluor R488 goat-anti-rabbit IgG (H+L) were used in the experiment. Parasites were frozen in Tissur-Tek O.C.T. Compound and cryosections were cut for immunohistochemistry. Localization of Em-TSP1 and Em-TSP3 in cysts (A), protoscoleces (B), adult worms (C) and eggs (D). The scale of microscopy images is shown on the lower right of each slice (scale bar = 100 µm in (A)–(C) and 25 µm in (D)). Fluorescence-labeled Em-TSP1 and Em-TSP3 on the surface of cysts, protoscoleces and adults are indicated by the red arrows. Expression of Em-TSP3 on the sucker and rostellum of the protoscoleces and on the oncospheres of eggs is indicated by the blue arrows. Pre-immunized serum (negative control) had no obvious reaction with any of fixed tissue sections. Microscopic images of HE-stained larvae, adults and eggs are shown as a reference. GL = geminal layer; PSC = protoscolex; HT = host tissue; T = tegument; R = rostellum; S = sucker; E = egg; O = oncosphere; and M = membrane.

Staining revealed immunolocalization of Em-TSP1 and Em-TSP3 at the surface (germinal layer/tegument) of both forms of *E. multilocularis* larva (cyst and protoscolex) (Figure 1A,B) and both of the antigens were also detected on the tegument of the adult worms (Figure 1C). Interestingly, expression of Em-TSP3 was also detected on the sucker and rostellum of the protoscoleces (Figure 1B) and on the oncospheres in adult eggs (Figure 1C,D). Pre-immunized serum, used as a negative control, had no obvious reaction with any of the fixed tissues (Figure 1). Microscopic images of H&E-stained larvae, adults and eggs are also shown as a reference (Figure 1).

### Determination of rEm-TSP1 and -TSP3 vaccine efficacies after i.n. administration

In experiment I, vaccination of mice with rEm-TSP3 plus CpG significantly reduced the liver cyst lesion numbers (62.1%, *p*<0.001), compared with that in the PBS control group. The liver CLNR after immunization with rEm-TSP1 plus CpG was significant (*p*<0.01), but lower (37.1%) than that after immunization with rEm-TSP3. A mixture of rEm-TSP1 and -TSP3 also resulted in a significant, but lower (28.7%, *p*<0.05), rate of liver CLNR than either rEm-TSP1 or rEm-TSP3. The CLNRs in the CpG and TRX control groups were 10.9% and 22.1% (*p*>0.05), respectively (Table 3). The difference in the vaccine efficacy between rEm-TSP1 and -TSP3 was statistically significant (*p*<0.05).

**Table 3:** Cyst lesion numbers and reductions achieved in experiment I.

Group	CLN		CLNR	CLRR (%)
	Range	Mean		
1 (PBS)	67–96	76.0	0	0
2 (PBS+CpG)	55–77	67.7	8.3	10.9
3 (TRX+CpG)	49–67	59.2	16.8	22.1
4 (rEm-TSP1+CpG)	36–58	47.8	28.2	37.1
5 (rEm-TSP3+CpG)	19–44	28.8	47.2	62.1

CLN = Cyst lesion number;

CLNR = Cyst lesion number reduction;

CLRR = Cyst lesion reduction rate.

### Vaccine efficacies of rEm-TSP3 administered via different routes

In experiment II, s.c. immunization with rEm-TSP3 significantly reduced the number of cyst lesions (82%, *p*<0.001) compared with that in the control group. The liver CLNR in the TRX and CpG control groups were 44% (*p*<0.05) and 27% (*p*<0.05), respectively. I.n. immunization with rEm-TSP3 plus CpG resulted in a CLNR of 61% (*p*<0.01), whereas rEm-TSP3 alone, and the TRX and CpG controls showed reductions of 22% (*p*>0.05), 38% (*p*<0.05) and 37% (*p*<0.05), respectively (Table 4). There was a significant difference between the vaccine efficacies of the two administration routes (*p*<0.05).

**Table 4:** Cyst lesion numbers and reductions achieved in experiment II.

Groups	CLN		CLNR	CLRR (%)
	Range	Mean		
1 (PBS)	47–74	56.4	0	0
2 (PBS+adj.)	29–50	41.0	15.4	27.3
3 (TRX+adj.)	11–58	31.6	24.8	44.0
4 (rEm-TSP3+adj.)	4–19	10.2	46.2	81.9
5 (PBS)	46–78	54.2	0	0
6 (PBS+CpG)	26–39	34.0	20.2	37.3
7 (TRX+CpG)	16–47	33.8	20.4	37.7
8 (rEm-TSP3)	29–62	42.2	12.0	22.1
9 (rEm-TSP3+CpG)	9–29	21.0	33.2	61.3

adj. = CFA/IFA adjuvant;

CLN = Cyst lesions number;

CLNR = Cyst lesion number reduction;

CLRR = Cyst lesion reduction rate.

### Antibody response in mice immunized s.c. and i.n. with rEm-TSP3

**Figure 2:**
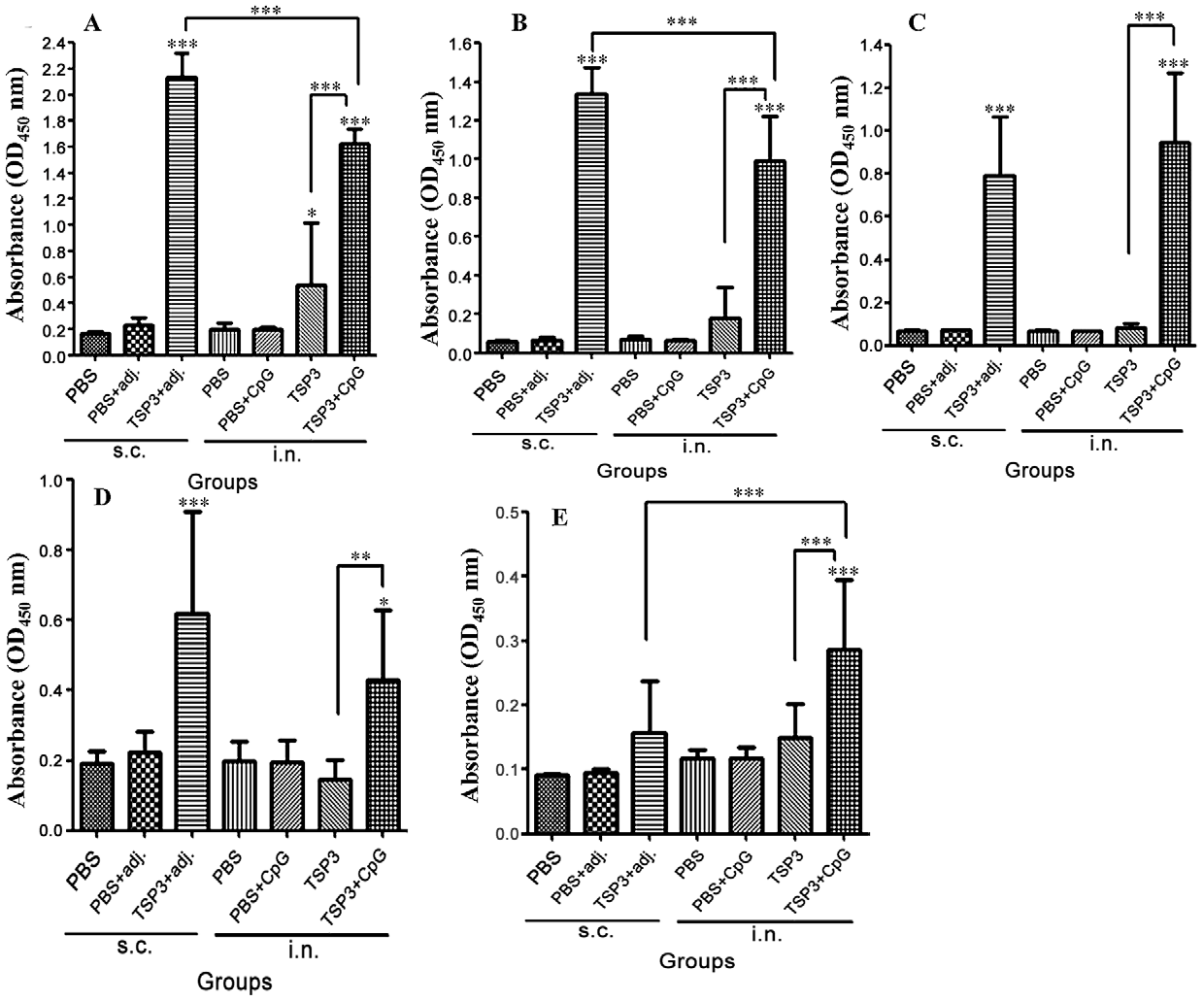
Serum antibody responses in BALB/c mice immunized with rEm-TSP3 either subcutaneously or intranasally. BALB/c mice were immunized with recombinant Em-TSP3 at a dose of 50 µg/mouse plus adjuvant (CpG for the intranasal group or Freund's complete adjuvant (CFA) or Freund's incomplete adjuvant (IFA) for the subcutaneous group). After the third immunization, blood was collected and sera IgG (A), IgG1 (B), IgG2α (C), IgM (D) and IgA (E) were assayed by ELISA. Results represent the mean absorbance measured at 450 nm for each group. Significant differences between the vaccinated groups and the PBS control group are denoted by an asterisk over the bar. Significant differences between any of the groups are denoted by an asterisk over the line connecting them. n = 8 per group; s.c. = subcutaneous; i.n. = intranasal; adj. = adjuvant IFA/CFA. *p<0.05 (significant); **p<0.01 (very significant); ***p<0.001 (extremely significant).

Antibody responses against rEm-TSP3 evoked by s.c. and i.n. administration were detected by ELISA. Compared with the PBS control, significant IgG responses were detected in the groups immunized with rEm-TSP3+CFA/IFA (*p*<0.001) and rEm-TSP3+CpG (*p*<0.001) (Figure 2A). A significant difference was observed between the two administration routes (*p*<0.001). In the i.n. group, a significant difference was observed between rEm-TSP3+CpG and rEm-TSP3 (*p*<0.001) alone. Strong IgG1 and IgG2α subclass antibody responses were induced by both s.c. and i.n. administration of rEm-TSP3 with adjuvants (*p*<0.001), while rEm-TSP3 alone induced neither significant IgG1 nor IgG2α responses (Figure 2B,C). Significant IgM antibody responses were detected in both s.c. (*p*<0.001) and i.n. (*p*<0.05) groups immunized with rEm-TSP3 plus adjuvant, but there was no significant difference observed between them. rEm-TSP3 alone did not induce a significant IgM antibody response (Figure 2D). A relatively strong serum IgA antibody response was detected in the group immunized with rEm-TSP3+CpG (*p*<0.001) (Figure 2E).

**Figure 3:**
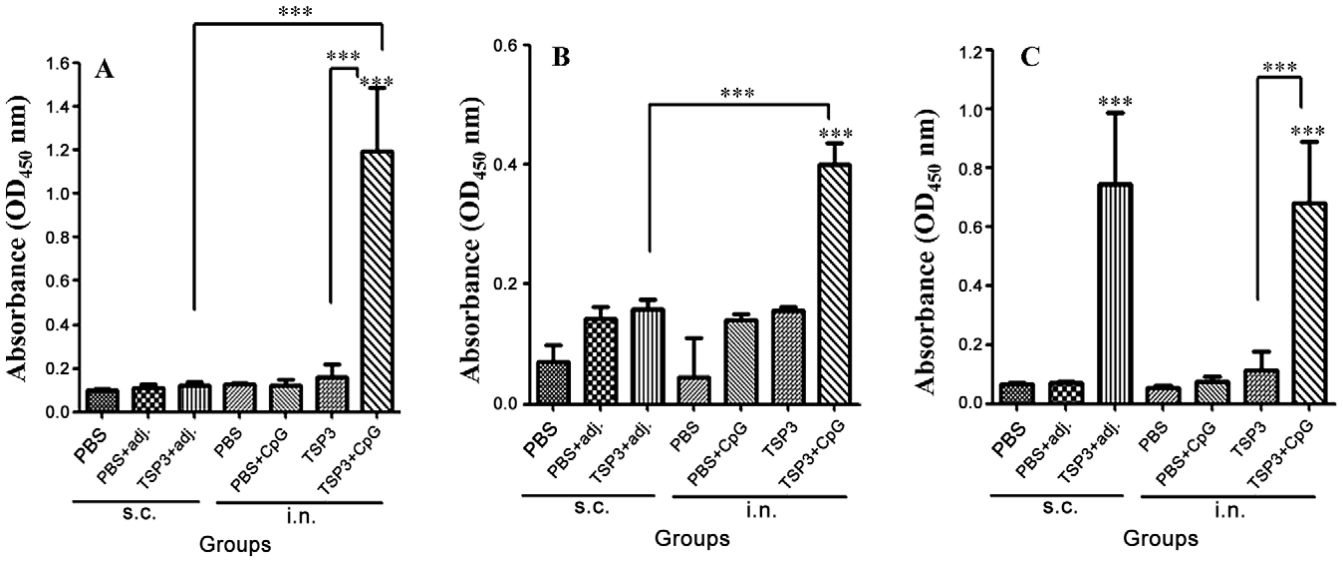
Local mucosal IgA responses induced in mice immunized with rEm-TSP3 either subcutaneously or intranasally. Three out of eight mice from each group were sacrificed and nasal washes, liver extracts and intestinal washes were collected for measurement of nasal IgA responses (A), intestinal IgA responses (B) and liver IgA responses (C) by ELISA. Significant differences between the vaccinated groups and the PBS control group are denoted by an asterisk over the bar. Significant differences between any of the groups are denoted by an asterisk over the line connecting them. n = 3 per group; s.c. = subcutaneous; i.n. = intranasal; adj. = adjuvant IFA/CFA. *p<0.05 (significant); **p<0.01 (very significant); ***p<0.001 (extremely significant).

In the nasal cavity, an extremely high IgA response (*p*<0.001) was only detected in the group i.n. immunized with rEm-TSP3+CpG (Figure 3A). I.n. immunization with rEm-TSP3+CpG induced higher intestinal IgA responses (*p*<0.001) than the other groups (Figure 3B). In the liver extracts, significant IgA responses were detected in the groups immunized with rEm-TSP3+CFA/IFA (s.c.) (*p*<0.001) and rEm-TSP3+CpG (i.n.) (*p*<0.01), with the former being slightly higher (Figure 3C).

Th1 and Th2 cell responses were assessed according to the IgG1/IgG2α ratio (Table 5). Two weeks post-immunization, s.c. immunization induced a mixed Th1/Th2 response, with the Th2 response predominating. This shifted to a reduced IgG1/IgG2α ratio 3 weeks post-immunization (a Th1 tendency). Inversely, i.n. immunization resulted in a tendency to shift from a Th1-dominant response to Th2-dominant one.

**Table 5:** IgG1 and IgG2α antibody profiles post-s.c. and -i.n. vaccination with rEm-TSP3.

Administration routes				Groups		
		IgG1		IgG2α		IgG1/IgG2α
		rEm-TSP3	PBS	rEm-TSP3	PBS	rEm-TSP3
s.c.	2 weeks p.i.	0.827±0.455	0.060±0.003	0.457±0.251	0.071±0.007	1.81
	3 weeks p.i.	1.297±0.124*	0.061±0.003	1.093±0.274*	0.070±0.004	1.19
i.n	2 weeks p.i.	0.344±0.098	0.065±0.019	0.432±0.148	0.071±0.005	0.80
.	3 weeks p.i.	1.014±0.233*	0.070±0.014	0.834±0.201*	0.068±0.003	1.22

s.c. = subcutaneous;

i.n. = intranasal;

p.i. = post-immunization;

*: Statistically significant compared with PBS control group (*p*<0.05).

### Complement-mediated lysis of protoscoleces

**Figure 4:**
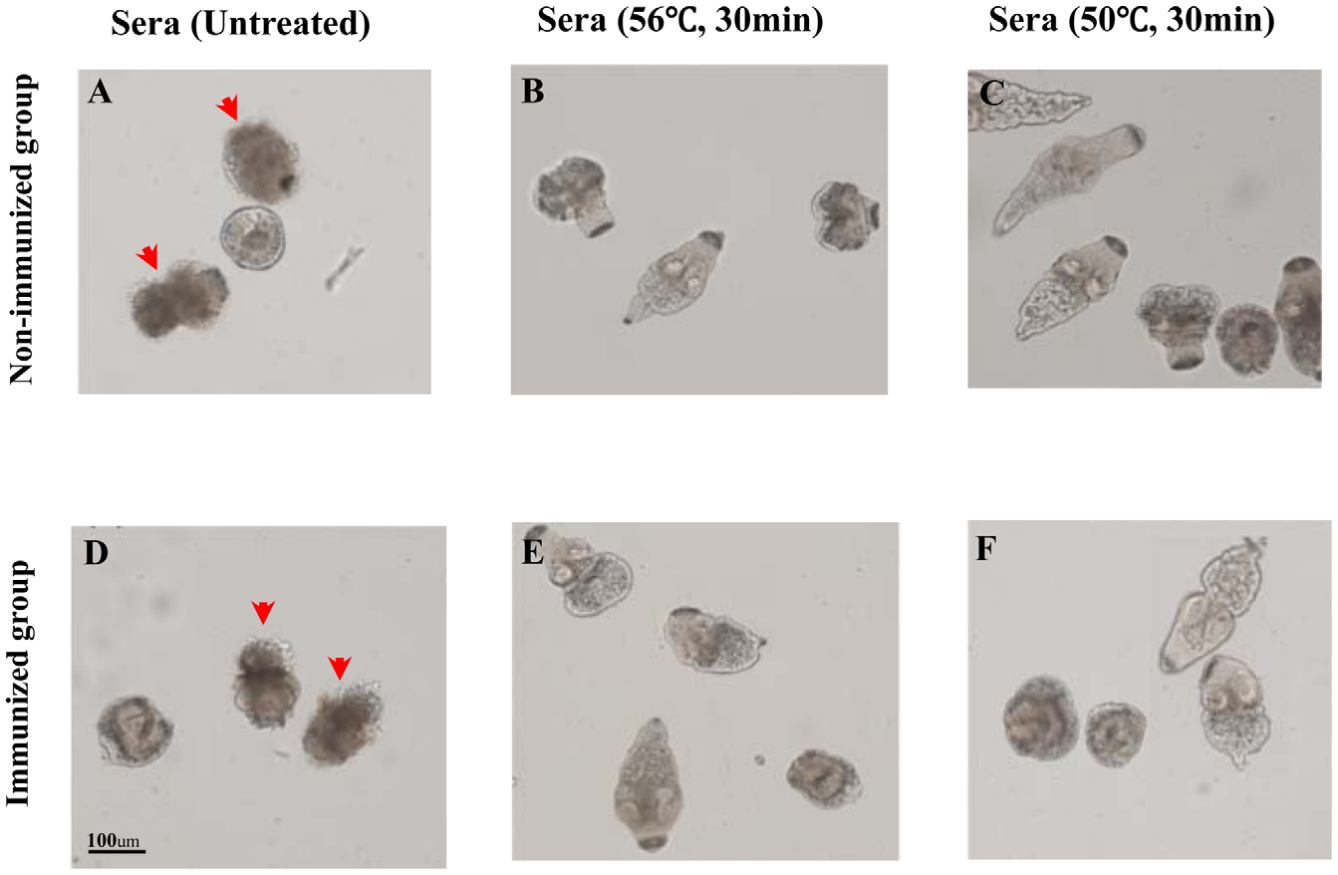
Complement-mediated lytic effects against protoscoleces. (A) and (D), lysis of protoscoleces by untreated/normal sera from non-immunized and rEm-TSP3-immunized BALB/c mice. (B) and (E), complement-inactivated sera (56°C for 30 min) did not lyse the protoscoleces. (C) and (F), no protoscolex lysis was evident after C3 proactivator in the serum was inactivated (50°C for 30 min). Lysed protoscoleces are indicated by a red arrow.

Complement-mediated lysis of protoscoleces began 2 h after the addition of untreated sera (from both non-immunized and immunized groups) and most were lysed at 8 h (Figure 4A,D); however, no lysis of protoscoleces treated with complement-inactivated sera (Figure 4B,E) was observed. Also, there was no visible protoscolex lysis after inactivation of C3 proactivator at 50°C for 30 min (Figure 4C,F).

## Discussion

As a transmembrane protein, tetraspanin is abundantly expressed on the tegument or body wall of some helminthes, including *Schistosoma mansoni* [15], [36], *S. japonicum* [16] and *Caenorhabditis elegans* [37], and is believed to play very important roles in signal transduction, cell proliferation, adhesion, migration, fusion and host-parasite interactions [38], [39]. Recently, we cloned seven tetraspanins (Em-TSP1–7) from *E. multilocularis* [18], of which only Em-TSP5 (E24) was confirmed its location on the surface of the larvae (cysts and protoscoleces) [40]. In this study, immunohistochemical analysis showed that both Em-TSP1 and -TSP3 are expressed on the surface of larvae and adult worms. Notably, the expression of Em-TSP3 on sucker, rostellum and inner tegument of protoscoleces and oncospheres was also observed. From these results we proposed that these proteins play important roles in *Echinococcus*-host interactions and that using them as vaccines may interfere with the parasite survival strategy [15], [17], [41]–[44]. Moreover, as reported previously, tetraspanins showed their potential protective effects against different stages of *Schistosoma* infections [15], [41]–[43]. Thus, vaccination of Em-TSP1 and -TSP3 proteins is believed to provide ‘broad-spectrum protection’ against the different stages infection by *E. multilocularis*.

We previously showed the high protective efficacy of seven tetraspanins (TSP1–7) against *E. multilocularis* infection in BALB/c mice after s.c. administration with Freund's adjuvant [18]. However, the toxicity of Freund's adjuvant limits the application of this vaccine model to human vaccines [19], [20]. Therefore, in this study, we used nontoxic CpG ODN as a vaccine adjuvant, which induces both systemic and mucosal immune responses in immunized animals [21], and evokes strong protective immune responses when used as mucosal adjuvant [22], [25], [45]–[47]. Studies on evaluation of CpG adjuvant efficient in primates (including humans) undoubtedly is an important step in human mucosal vaccine development [25], [26]. Recently, CpG was used as a mucosal adjuvant in developing anti-protozoan and -helminth vaccines [22]–[24]. Of the different mucosal administration routes, i.n. delivery is the most appropriate, mainly because it induces the full range of local immune responses (so-called ‘common mucosal immune system’) [28] and induces strong immunity after only small dose of vaccine [29], [30].

Based on the above properties, i.n. immunization and the CpG adjuvant has many advantages; therefore, we evaluated the efficacy of i.n. delivery of the rEm-TSP1 and -TSP3 vaccines using CpG as an adjuvant, and further investigated the systemic and mucosal immune responses mounted against rEm-TSP3, compared with those induced by s.c. immunization using Freund's adjuvant.

In experiment I, significantly high liver CLNRs, resulted from i.n. immunization with rEm-TSP1 plus CpG (37.1%, *p*<0.01) and rEm-TSP3 plus CpG (62.1%, *p*<0.001), were observed compared with the PBS control. However, our previous study reported the efficacies of 87.9% and 85.1%, respectively, for the rEm-TSP1 and -TSP3 vaccines, when administered s.c. with Freund's adjuvant [18]. Taken together, our own results and those of others [48], conclude that different antigen delivery routes greatly affect vaccine efficacy.

In experiment II, we focused on the rEm-TSP3 protein, which showed higher liver CLNR than rEm-TSP1 in experiment I. We investigated systemic and mucosal immune responses associated with protection in BALB/c mice immunized s.c. or i.n. Under the same conditions, the vaccine efficacies induced by the two immunization routes were similar to those observed in Experiment I and our previous study [18]. ELISA data showed that, after the third immunization, extremely significant serum IgG immune responses were induced by both the administration routes (*p*<0.001), with the former stronger than the latter (*p*<0.001). Meanwhile, significant levels of IgG1, IgG2α and IgM antibodies were detected. Th1 and Th2 cell responses were evaluated according to the IgG1/IgG2α ratio. After the second immunization, a mixed Th1/Th2 cell response was evoked in the s.c.-immunized group, dominated by a Th2 response, whereas the IgG1/IgG2α ratio reduced after the third immunization (a Th1 tendency). Inversely, i.n. immunization resulted in a shift from a Th1 to a Th2 response. As previously reported, antibodies form a critical part of the immune response against taeniid metacestodes, with IgG1, IgG2α, IgG2β, and IgE play a major role in oncosphere killing, although the involvement of other mechanisms can not be ruled out [4]. Antibody-dependent, complement-mediated lysis is a pivotal characteristic during the early stages of taeniid cestodes infection of intermediate hosts [4], [49]. Interestingly, our *in vitro* complement assay showed the lytic effect on protoscoleces by both normal/non-immunized and rEm-TSP3-immunized serum from BALB/c mice. Notably, treatment of sera at 56°C for 30 min (complement inactivation) or at 50°C for 30 min (C3 proactivator inactivation) abolished protoscolex lysis, suggesting that at least an alternative pathway exists in BALB/c mice for complement activation in the initial infection of *Echinococcus* metacestode.

Growing evidences suggest the importance of a Th1/Th2 balance during parasite infections, such as infections by *Trypanosoma* [50], *Schistosoma* [15], [17] and *Echinococcus* [4]–[8], [10]–[13]. In *Echinococcus* infection, early Th1-polarized cytokine production, which can kill the metacestodes at the initial stages of development, shifts to a Th2 cytokine response during the chronic stage [4], [6], [8]. The characteristic of Th1 profile, mainly induced by IL-12, is the secretion of IL-2, TNF and especially IFN-γ, which lead to the recruitment and activation of the cellular effector phase of immunity [51], [52]. A shift from a Th1 to a Th2 cytokine profile, mainly induced by an increased secretion of IL-10, a cytokine typically associated with immunoregulation of effector responses, is thought to limit and ultimately terminate inflammatory responses [53]–[55].

*Echinococcus* metacestode has developed a number of strategies for escaping host immune attack [4]–[7]. The shift from a protective immune response (Th1 response) to a nonprotective one (Th2 response) is thought to be one of the most important mechanisms, whereby *Echinococcus* metacestodes regulate host immune responses to benefit their long-term survival in the hosts, by using some molecules like antigen B [56]. Tetraspanins in the tegument of schistosomula and adult worms are suggested to act as receptors for host ligands, including MHC molecules, by which parasites mask their nonself status, and thereby escape host immune responses [15]. In the present study, because CpG was expected to (but actually failed to) elicit significant Th1 cell responses as previously reported [21], we hypothesize that the tetraspanins used also share the same immunomodulatory mechanism as antigen B. This would offset of the adjuvant activity of CpG (and even Freund's adjuvant), which would provide a reasonable explanation for the induction of predominant Th2 cell responses in this study.

It is clear that systemic immune responses, as mentioned above, play a crucial role in protection against *Echinococcus* parasite infections. However, since the early, natural infection of eggs/oncospheres begins at the gastrointestinal membrane and the invasion, rooting and development/proliferation of *Echinococcus* metacestodes occurs in the host organs (mainly the liver), the immunological events occurring at the local mucosa should not be neglected.

Secretory IgA is a critical component of the mucosal immune system and plays an important role as the first lines of defense against many parasite infections, such as Giardia [57], [58] and *Echinococcus* [59]. IgA responses in the mucosa might be hypothesized to target the parasite by neutralizing parasite ES products, attenuating the parasite-host interaction and interfering with parasite feeding and survival [60]–[62]. Induction of eosinophil degranulation by IgA is another important characteristic of mucosal immunological events as observed by Abu-Ghazaleh *et al.* [63]. Additionally, mucosal immunity could play a role in tolerance induction against *E. multilocularis* that may be a prerequisite for the subsequent development of the larvae in the liver, and for the occurrence of alveolar proliferation [59].

In the present study, a remarkably strong intranasal IgA response was induced (*p*<0.001) even though only a very low level of serum IgA was induced, by i.n. immunization with rEm-TSP3 plus CpG; however, only a limited intestinal IgA response was detected. rEm-TSP3 alone (without any adjuvant) failed to induce a high IgA response in either i.n.-or s.c.-immunized groups. It is noteworthy that high levels of IgA antibodies were detected in liver extracts after immunization by both immunization routes. Although the exact mechanisms underlying IgA anti-parasite response in the liver are unknown, infection of this organ by *E. multilocularis* larvae is characterized by a chronic process; antibody responses in the liver, including IgA, as well as other immune-associated factors should not be neglected [6], [64], [65] and further studies regarding their roles are required.

Taken together, the results of the present study suggest that i.n. administration of rEm-TSP3 plus CpG, which induces systemic and local immune responses, is a prospective model for the development of nontoxic human vaccines. It is also proposed that, in our vaccine model, protection is mainly provided by serum antibodies including antibody-dependent, complement-mediated lysis against early infection by *E. multilocularis* (oncospheres in blood). Although intestinal IgA is believed to play an important role in inhibiting invasion of hatched oncospheres, our vaccines did not induce satisfactory levels of IgA antibodies; this will be an important consideration in our further work. Moreover, the extensive expression of Em-TSP3 on the surface/tegument of *E. multilocularis* at different developmental stages suggests the possibility of developing TSP-based vaccines with ‘broad-spectrum protection’ against the worm infections at different stages (oncosphere, cyst, protoscolex and even adult). On the other hand, the surface/tegument tetraspanin proteins may have crucial functions in protecting the *Echinococcus* parasite by shifting the host protective immune response (Th1 response) to non-protective one (Th2 response), especially during the stages of liver infection. Therefore, further work is needed to develop new strategies for offsetting this ‘undesired effect’ of tetraspanin proteins, such as enhancement of the biological activity of the CpG ODN adjuvant by modifying its backbone chemistry, and the use of different delivery methods, including mixing or cross-linking of CpG ODN to other carrier compounds as reviewed by Mutwiri *et al.*, elsewhere [66].

## Supporting Information

Figure S1 **Classical morphology of an egg from the feces of an experimentally-infected dog.** E = embryophore; O = oncosphere; H = hook.
(TIF)

Figure S2 **Cyst lesions formed by** ***E. multilocularis*** **larvae in BALB/c mice liver.** Immunized mice were anesthetized and challenged orally with 200 *E. multilocularis* eggs. One month post-infection, all mice were sacrificed and the livers were collected and cut into slices to count the number of cyst lesions. Cysts are marked in blue circles.
(TIF)

Table S1 **List of Genbank accession numbers for the genes referred to in the text.**
(DOC)
