# Supplementary figures and images for: A Pilot Study on Developing Mucosal Vaccine against Alveolar Echinococcosis (AE) Using Recombinant Tetraspanin 3: Vaccine Efficacy and Immunology
Source: PLoS Negl Trop Dis. 2012 Mar 27;6(3):e1570. doi: 10.1371/journal.pntd.0001570 (PMC3313938; doi:10.1371/journal.pntd.0001570)

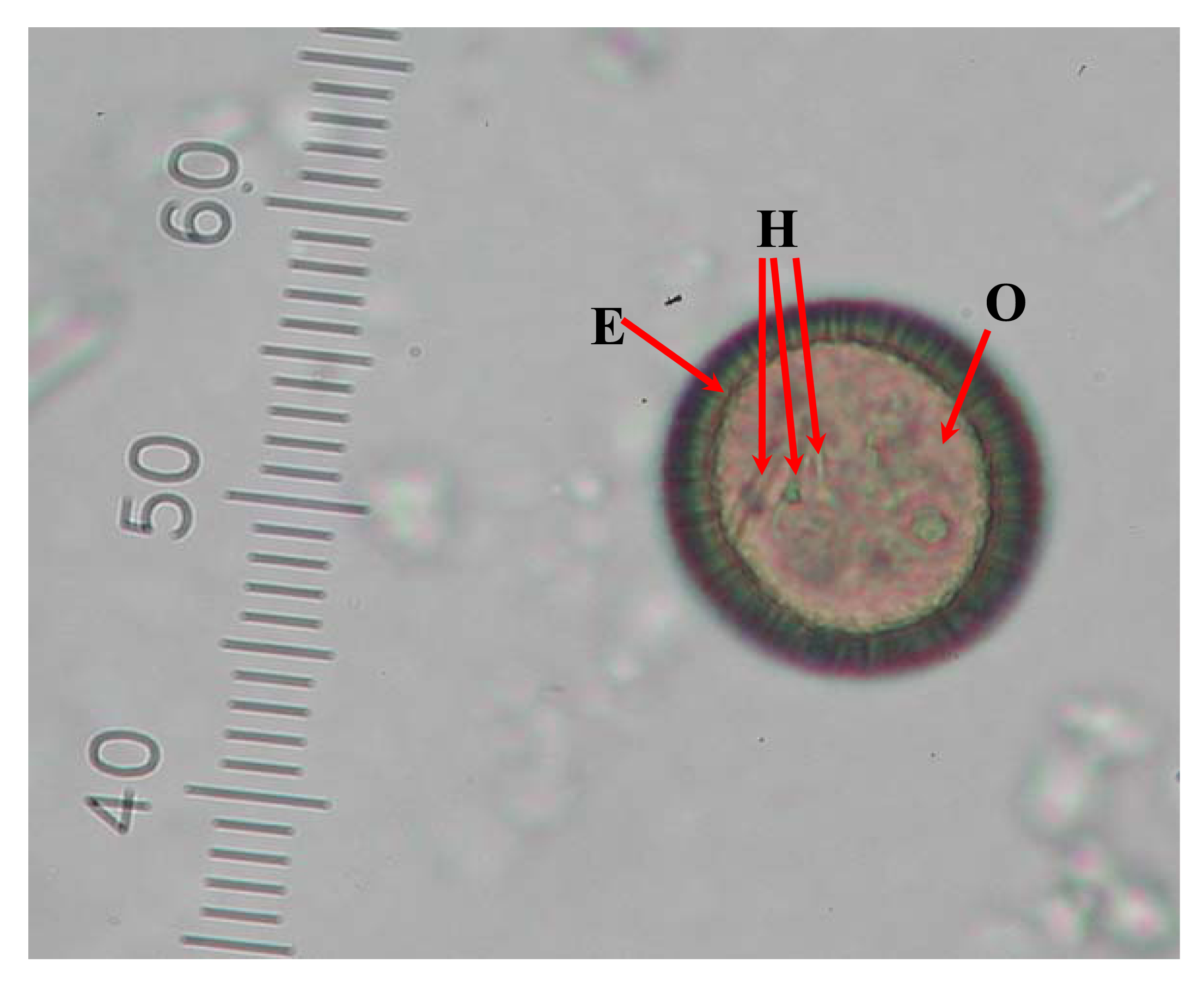

Supplement: Figure S1 — Classical morphology of an egg from the feces of an experimentally-infected dog. E = embryophore; O = oncosphere; H = hook. (TIF) [file pntd.0001570.s001.tif]

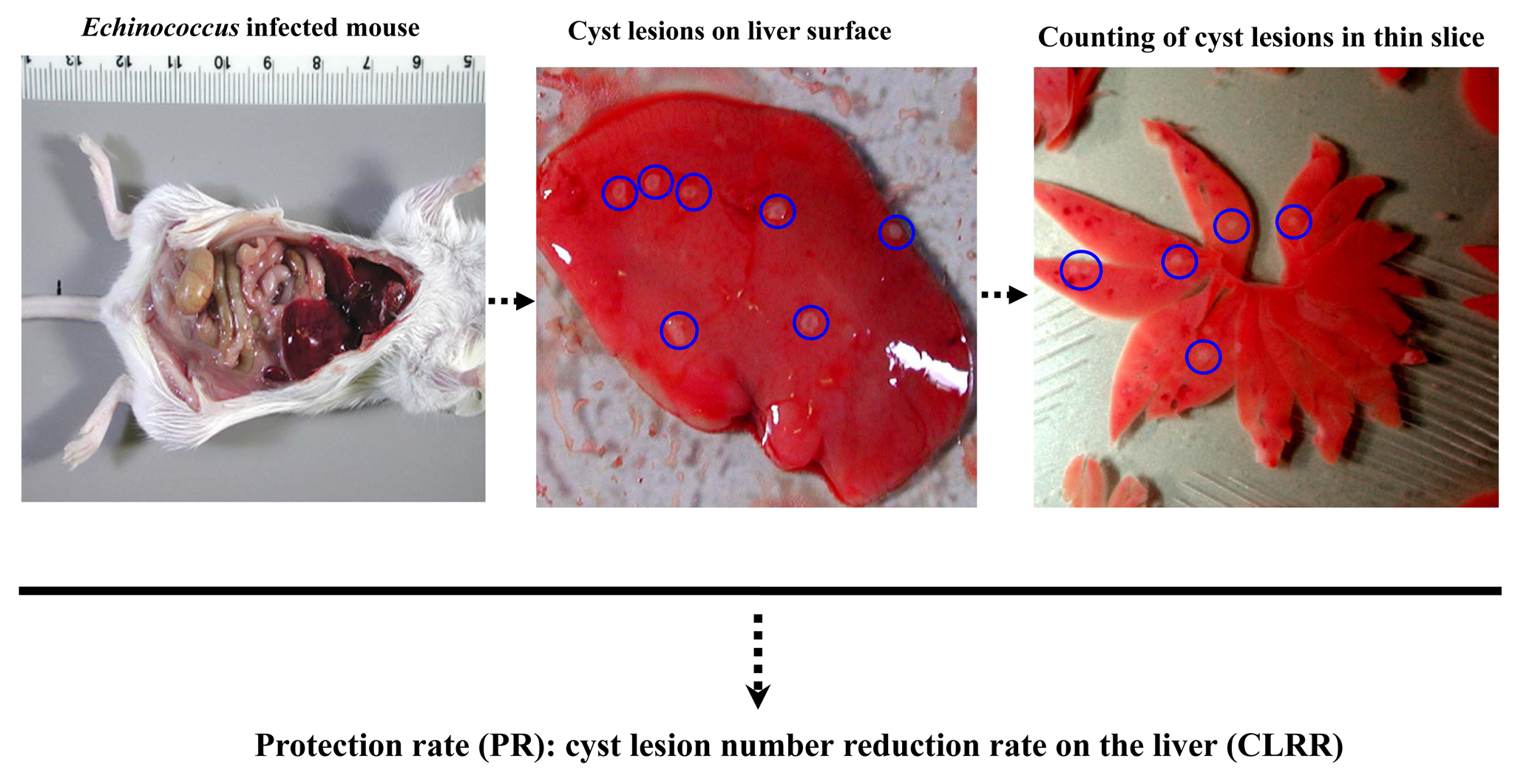

Supplement: Figure S2 — Cyst lesions formed by E. multilocularis larvae in BALB/c mice liver. Immunized mice were anesthetized and challenged orally with 200 E. multilocularis eggs. One month post-infection, all mice were sacrificed and the livers were collected and cut into slices to count the number of cyst lesions. Cysts are marked in blue circles. (TIF) [file pntd.0001570.s002.tif]
