# Supplementary material for: A Pilot Study on Developing Mucosal Vaccine against Alveolar Echinococcosis (AE) Using Recombinant Tetraspanin 3: Vaccine Efficacy and Immunology
Source: PLoS Negl Trop Dis. 2012 Mar 27;6(3):e1570. doi: 10.1371/journal.pntd.0001570 (PMC3313938; doi:10.1371/journal.pntd.0001570)
Supplement: Table S1 — List of Genbank accession numbers for the genes referred to in the text. (DOC) [file pntd.0001570.s003.doc]

| **Gene** | **Parasite** | **Accession number** | **Reference** |
| --- | --- | --- | --- |
| Sm-TSP-1 | *Schistosoma mansoni* | AF521093 | 15 |
| Sm-TSP-2 | *S. mansoni* | AF521091 | 15 |
| Sm29 | *S. mansoni* | AF029222 | 17 |
| Em-TSP1 | *Echinococcus multilocularis* | FJ384717.1 | 18 |
| Em-TSP2 | *E. multilocularis* | FJ384718.1 | 18 |
| Em-TSP3 | *E. multilocularis* | FJ384719.1 | 18 |
| Em-TSP4 | *E. multilocularis* | FJ384720.1 | 18 |
| Em-TSP5 | *E. multilocularis* | FJ384721.1 | 18 |
| Em-TSP6 | *E. multilocularis* | FJ384722.1 | 18 |
| Em-TSP7 | *E. multilocularis* | FJ384716.1 | 18 |
| AS14 | *Ascaris suum* | AB057441 | 34 |
| AS16 | *A. suum* | AB089179 | 35 |
| 43-KDa antigen | *Trichinella spiralis* | M95499 | 36 |
| 22-KDa antigen | *S. mansoni* | M37003 | 39 |
| TSP15 | *Caenorhabditis elegans* | MM_060003 | 42 |
| Sm23 | *S. mansoni* | L34755 | 46, 48 |
| Sj23 | *S. japonicum* | M63706 | 47, 48 |
| TcTs | *Trypanosoma cruzi* | L26499 | 49 |
| ASP-2 | *T. cruzi* | U77951 | 49 |
| gp63 | *Leishmania donovani* | GQ301544 | 51 |
| TcG1 | *T. cruzi* | AY727914 | 56 |
| TcG2 | *T. cruzi* | AY727915 | 56 |
| TcG4 | *T. cruzi* | AY727917 | 56 |
| EgAgB | *E. granulosus* | M36774 | 62, 63 |

**Table S1**
